# Dysbiosis-Mediated Regulation of Stem Cells the First Hit for Cancer Generation

**DOI:** 10.3390/ijms27020628

**Published:** 2026-01-08

**Authors:** Ciro Gargiulo-Isacco, Van Hung Pham, Kieu C. D. Nguyen, Toai C. Tran, Sergey K. Aityan, Raffaele Del Prete, Emilio Jirillo, Luigi Santacroce

**Affiliations:** 1 Department of Interdisciplinary Medicine, Section of Microbiology and Virology, School of Medicine, University of Bari “Aldo Moro”, 70124 Bari, Italy; drkieukaren@gmail.com (K.C.D.N.); raffaele.delprete@uniba.it (R.D.P.); emilio.jirillo@uniba.it (E.J.); luigi.santacroce@uniba.it (L.S.); 2 Viet Nam Research and Development Institute of Clinical Microbiology, Ho Chi Minh City 756000, Vietnam; phhvan.nkbiotek@gmail.com; 3 Basic Medical Sciences Faculty, Pham Ngoc Thach University of Medicine, Ho Chi Minh City 740500, Vietnam; 4 Department of Multidisciplinary Engineering, Northeastern University, Oakland Campus, Oakland, CA 94613-1301, USA; s.aityan@northeastern.edu

**Keywords:** cancer, dysbiosis, microbiota, hormones, cancer stem cells, immunity, β-glucuronidase (GUS)

## Abstract

Human microbiota, a complex consortium of microorganisms co-evolved with the host, profoundly influences tissue development, immune regulation, and disease progression. Growing evidence shows that microbial metabolites and signaling molecules modulate key stem cell pathways—such as *Hedgehog*, *Wnt/β-catenin*, and *Notch*—thereby reprogramming stem cell fate toward tumor-suppressive or tumor-promoting outcomes. Specific taxa within oral, intestinal, and urogenital niches have been linked to cancer initiation, therapy resistance, and recurrence. In parallel, clinical studies reveal that microbiota composition affects infection dynamics: bacterial isolates from symptomatic urinary tract infections inhibit commensal growth more strongly than the reverse, with Gram-positive and Gram-negative strains displaying distinct interaction profiles. Collectively, these findings highlight microbiota’s dual role in regulating cellular plasticity and pathogenicity. Elucidating host–microbe and microbe–microbe mechanisms may guide microbiota-targeted interventions to improve cancer and infectious disease management.

## 1. Microbiota, Age and Cellular Reprogramming in Health and Disease

### Introduction

Despite the central role of genetic mutations in classical models of carcinogenesis, these frameworks remain mostly incomplete. They do not fully explain the spatially and temporally extensive tissue vulnerability observed prior to malignant transformation, nor do they account for why genetically similar epithelia often exhibit highly divergent cancer risk [1]. These limitations suggest the existence of upstream, non-genetic determinants that shape the carcinogenic landscape before irreversible genomic alterations occur [1].

Accumulating evidence supports a model in which aging-related factors (including stem cell dysfunction, hormonal imbalance, and microbial dysbiosis) play a critical upstream role in cancer susceptibility [2]. Aging, combined with microbial dysbiosis, represents a persistent and hushed perturbation of the mucosal microenvironment, capable of durably altering metabolite availability, microbial enzymatic activity, immune tone, and epithelial signaling [3]. As such, dysbiosis is uniquely positioned to act upstream of subsequent genetic damage, reshaping tissue homeostasis long before malignant transformation becomes detectable [3].

In this context, a “first hit” does not indicate an initiating mutation but rather a permissive, ongoing, and often clinically silent process that disrupts epithelial homeostasis by impairing stem-cell quiescence, niche signaling, and regenerative fidelity. This disruption lowers the threshold for subsequent genetic or epigenetic alterations [4,5]. The concept of field cancerization provides a valuable framework for understanding how widespread biological perturbations predispose large epithelial regions to malignant progression [5].

Beyond compositional shifts, dysbiosis alters the functional output of the microbiome, including microbial enzymatic activities that regulate the local bioavailability of xenobiotics, host-derived metabolites, and hormones essential for immune regulation and epithelial maintenance [6,7]. Of particular relevance are glucuronide-processing pathways and steroid hormones such as testosterone, estrogen, and progesterone, whose dysregulation can lead to localized reactivation of bioactive compounds. This, in turn, exposes epithelial cells and stem-cell niches to chronic metabolic stress that promotes tumor-permissive microenvironmental remodeling [6,7,8].

The human microbiota is a highly diverse and dynamic consortium of microorganisms that has co-evolved with its host since the earliest stages of human evolution and exerts profound effects on tissue development, immune homeostasis, and regenerative capacity [9,10,11]. Increasing evidence indicates that microbial communities influence both normal and cancer stem cell biology through complex metabolic and molecular interactions [11,12,13]. The microbial microenvironment, including metabolites, inflammatory and anti-inflammatory mediators, and diverse signaling molecules, modulates key stem cell pathways such as *Hedgehog*, *Wnt/β-catenin*, and *Notch*, as well as apoptosis-related cascades involving STAT1–3, SMAC/DIABLO, and HIF-1 [4,5]. These interactions can reprogram stem cell fate toward tumor-suppressive or tumor-promoting outcomes, reactivate multipotency genes such as *Nanog*, *Sox2*, and *Oct4*, and thereby shape self-renewal, differentiation, and malignant transformation [4,5].

Furthermore, the microbiota has emerged as a critical determinant of the efficacy and toxicity of cell-based anticancer therapies, given its capacity to regulate stem cell plasticity, immune signaling, and metabolic adaptation [8]. Specific bacterial taxa have been associated with distinct malignancies; for example, components of the oral microbiota can activate inflammatory and cancer stem cell-related pathways in head and neck cancers, glioblastomas, and lung carcinomas [9,10], whereas dysbiosis of the intestinal and urogenital microbiota has been linked to colorectal, cervical, bladder, bone, and prostate cancers [11,12,13]. Cancer stem cells display intrinsic resistance to chemotherapy, and a dysbiotic microenvironment may further exacerbate this resistance by sustaining stemness-associated signaling programs and promoting tumor recurrence [14,15].

**Figure 1:**
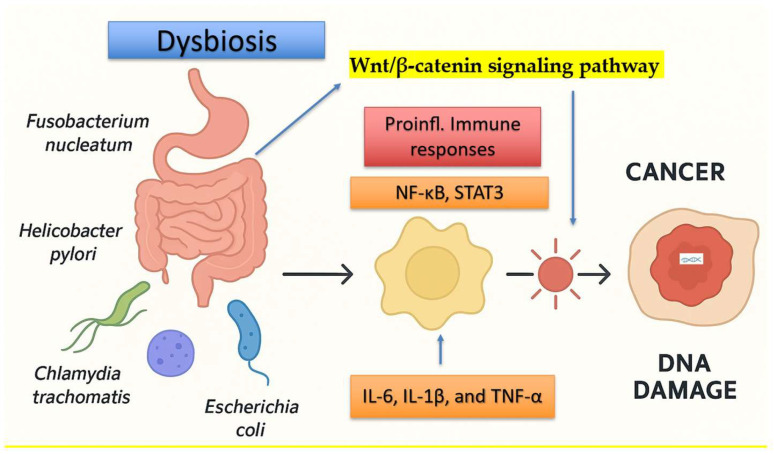
Schematic representation of the direct and indirect mechanisms by which oncogenic microbial species contribute to tumor initiation and progression. Pathogenic bacteria (e.g., *Fusobacterium nucleatum*, *Helicobacter pylori*, *Escherichia coli*, and *Chlamydia trachomatis*) induce epithelial DNA damage through the production of genotoxins, reactive oxygen species, and virulence factors, while simultaneously activating innate and adaptive immune responses. Chronic immune activation leads to sustained inflammatory signaling (e.g., NF-κB, STAT3, inflammasome pathways), resulting in cytokine release, oxidative stress, immune tolerance, and genomic instability that collectively promote malignant transformation.

In this work, we propose that microbial dysbiosis, acting in concert with the aging process, constitutes an early, non-genetic “first hit” in carcinogenesis by remodeling the epithelial tissue microenvironment, destabilizing stem-cell homeostasis, and generating preneoplastic fields that facilitate subsequent oncogenic events. Unlike prior reviews that primarily focus on associations between microbiome composition and cancer risk, this framework positions aging and dysbiosis as upstream, mechanistic determinants of tissue vulnerability. Importantly, the model generates testable predictions, including the expectation that microbiome-driven metabolic and enzymatic alterations may precede detectable genetic lesions and that restoration of microbial homeostasis may reverse early epithelial dysfunction before malignant transformation occurs [14,15]. A schematic overview of this dysbiosis-based model of carcinogenesis is provided in Figure 1.

## 2. Microbiota as an Autonomous Endocrine, Metabolic, Immune Organ

The intestinal and urogenital microbiota together can be regarded as a functional, autonomous organ, a complex, semi-independent system essential for nutrient processing and immune–endocrine regulation that is fundamental to host physiology [16]. Although anatomically distinct, the gut and urogenital microbial ecosystems may exert complementary and tightly interconnected functions. Through the coordinated production of bioactive metabolites acting as signaling molecules, these microbial communities operate as an integrated metabolic and regulatory network capable of profoundly influencing host metabolism, immune homeostasis, and cellular reprogramming [16].

Within the gut, microbial fermentation of indigestible carbohydrates produces short-chain fatty acids (SCFAs), including acetate, propionate, and butyrate, which serve as key energy substrates and signaling molecules with anti-inflammatory and antitumor properties [17]. Gut microbes synthesize essential vitamins, including folate and other B-group vitamins, and facilitate the intestinal absorption of key minerals such as iron, calcium, and magnesium. In addition, the intestinal microbiota contributes to host neuroendocrine–immune communication through the production and modulation of bioactive compounds, including serotonin and estrogen-like metabolites, thereby influencing systemic metabolic regulation [18].

Furthermore, microbiota-encoded carbohydrate-active enzymes (CAZymes) regulate the fermentation of dietary substrates and modulate intestinal hormone secretion, contributing to energy homeostasis, glucose metabolism, and appetite control [18]. Specific bacterial species have the potential distinct metabolic signatures. *Akkermansia muciniphila* degrades mucins while contributing to the production of propionate and butyrate, thereby reinforcing intestinal barrier function [19]. *Clostridium sporogenes* converts tryptophan to indolepropionic acid, a microbial metabolite with potent antioxidant activity that also inhibits tryptophan biosynthesis in *Mycobacterium tuberculosis* [20]. In the cecum, *Clostridium* species belonging to the phylum *Firmicutes* catalyze the conversion of primary to secondary bile acids—deoxycholic and lithocholic acids—through 7α-dehydroxylation [21]. These reactions influence nuclear farnesoid X receptor (FXR) signaling and bile acid synthesis, thereby modulating lipid and glucose metabolism [22]. Moreover, the gut microbiota synthesizes polyamines (putrescine, spermidine, spermine) via decarboxylation of ornithine, arginine, and lysine, supporting epithelial proliferation and mucosal repair [23].

By contrast, the genitourinary microbiota, especially the vaginal ecosystem dominated by *Lactobacillus* species, serves primarily as a barrier organ. It maintains an acidic pH, produces bacteriocins, and prevents colonization by opportunistic pathogens [24]. Although less involved in nutrient metabolism, the genitourinary microbiota exerts crucial immunomodulatory and protective functions. Importantly, many pro-oncogenic or opportunistic bacterial strains originate in the gastrointestinal tract and migrate to the urogenital region, establishing a functional continuum between these systems [25].

## 3. Microbial Interactions and Pathogenic Dynamics

In addition to its influence on metabolism and carcinogenesis, clinical studies have shown that the microbiota modulates infectious disease outcomes by directly or indirectly affecting bacterial pathogenicity [26,27]. Within the intestinal and urogenital tracts, resident microbial communities interact with invading pathogens through mechanisms that are not yet fully understood. However, recent evidence indicates that bacterial isolates from individuals with urinary tract infection (UTI)-like symptoms and those from asymptomatic subjects can reciprocally influence each other’s growth dynamics [28]. Isolates from symptomatic patients tend to exert stronger inhibitory effects on commensal strains than vice versa. Furthermore, Gram-positive and Gram-negative bacteria display distinct interbacterial communication patterns, reflecting fundamental differences in cell wall structure and quorum-sensing mechanisms [29,30].

Recent studies investigating the role of the intestinal microbiota in the etiopathogenesis of colon cancer have highlighted how specific microbial communities and their metabolites can contribute to tumor initiation and progression. This now widely accepted view proposes that the microbiome acts through multiple mechanisms, including the induction of DNA damage, activation of pro-oncogenic signaling pathways, modulation of epithelial metabolism, and regulation of local and systemic immune responses [29,30]. Collectively, these interactions reshape the tumor microenvironment, promoting chronic inflammation and immunosuppression, and positioning the microbiota as a promising target for preventive and therapeutic strategies in colorectal cancer [29].

This body of work reinforces a paradigm shift in which colorectal cancer is no longer viewed solely as a genetic disease but rather as a host–microbiome ecosystem disorder, shaped by long-term microbial–host coevolution and environmental influences. Such microbial interactions play a crucial role in maintaining mucosal ecological balance and may ultimately influence both susceptibility to infection and therapeutic outcomes [29,30].

## 4. Dysbiosis-Driven Carcinogenesis: Mechanistic Pathways

Dysbiosis of the intestinal and urogenital microbiota has been increasingly associated with the onset and progression of malignancies in multiple organs, including the gastrointestinal tract, bladder, prostate, cervix, breast, and endometrium [31,32,33]. This process is mediated by several interrelated mechanisms involving chronic inflammation, the production of carcinogenic metabolites, genotoxicity, and disruption of the immune–endocrine balance. This microenvironment is characterized by, (i) a substratum overrepresented by pathogenic species, that induce persistent inflammatory responses branded by elevated cytokines (e.g., IL-6, IL-1β, IL-17, and TNF-α), and an excessive generation of reactive oxygen species (ROS). These mediators cause continuous nuclear DNA damage and a metabolic shift toward aerobic glycolysis, creating the conditions conducive to neoplastic transformation and proliferation [34,35,36]. (ii) Production of carcinogenic metabolites, certain bacterial taxa produce oncogenic compounds such as N-nitrosamines and specific genotoxins. *Bacteroides fragilis* toxin and the *pks*^+^ *Escherichia coli* colibactin, may lead to DNA strand breaks, mutations, and genomic instability. In addition, some pathogens rely on their capability of subverting the apoptotic mechanisms such as the Smac/Diablo, HIF-1 and Stat-1 [37,38,39]. (iii) Immune–endocrine modulation, the microbiota profoundly influences the host’s immune surveillance and hormonal signaling. Dysbiosis can impair tumor immunoediting and alter the pituitary–adrenal axis, resulting in dysregulated secretion of estrogen, cortisol, progesterone, and testosterone. These hormonal imbalances can compromise immune defense mechanisms, easing tumor initiation and progression [40,41,42,43]. (iv) Disruption of the epithelial barrier and alterations in microbial composition impair epithelial integrity and mucosal tight junctions, facilitating bacterial translocation and the systemic dissemination of microbial metabolites and inflammatory mediators. This breach promotes chronic low-grade systemic inflammation and may contribute to carcinogenesis at distant sites, including extraintestinal and extra-urogenital tissues [44,45,46,47].

Therefore, the relationship between the human microbiome and cancer development represents one of the most complex and extensively investigated areas of contemporary biomedical research. Many of these bacteria are essential components of a healthy gut microbiome, such as *Escherichia coli*, which is an essential part of a healthy gut microbiome; however, some strains can produce more lethal toxins than others, which are capable of altering DNA [48]. Among these, associations between microbial dysbiosis and colorectal, gastric, cervical, uterine, prostate, bladder, and breast cancers are the most comprehensively characterized [48,49,50,51]. The host immune system responds to microbiota-derived metabolites by generating genotoxic and pro-inflammatory mediators, which can induce both nuclear DNA (nDNA) and mitochondrial DNA (mtDNA) damage, sustain chronic inflammation, and ultimately promote tumorigenesis [52,53]. This reciprocal interaction between microbial metabolites and host immune responses contributes to the initiation and progression of these malignancies. Specific bacterial species, including *Fusobacterium nucleatum*, *Helicobacter pylori*, *Chlamydia trachomatis*, and certain strains of *Escherichia coli* have been potentially implicated in the pathogenesis of these cancer types (Table 1) [54,55,56,57].

## 5. Microbiota and Specific Cancer Types

### 5.1. Colorectal Cancer (CRC)

Among all human malignancies, CRC certainly shows the strongest and most consistent association with gut dysbiosis. Several microbial pathogens have been identified as key contributors to tumor initiation and progression. Among these, *Fusobacterium nucleatum* is one of the most consistently associated species and promotes carcinogenesis through multiple interconnected mechanisms. These include activation of the Wnt/β-catenin signaling pathway via binding of its adhesin FadA to epithelial E-cadherin, recruitment and polarization of pro-tumorigenic myeloid cells, and modulation of the immune microenvironment leading to impaired cytotoxic T-cell-mediated antitumor responses [58,59]. In addition, *F. nucleatum* induces persistent activation of inflammatory signaling pathways, including NF-κB, STAT3, and inflammasome-associated cascades, resulting in sustained production of pro-inflammatory cytokines such as IL-6, IL-1β, and TNF-α [58,59]. This chronic inflammatory milieu possibly promotes epithelial stress, oxidative DNA damage, immune tolerance, and the activation of stemness- and EMT-related transcriptional programs, thereby facilitating tumor growth, progression, and resistance to immune surveillance [58,59]. Similarly, enterotoxigenic *Bacteroides fragilis* (ETBF) promotes epithelial transformation through the action of its toxin BFTs, which cleaves E-cadherin, disrupts epithelial integrity, activates β-catenin and NF-κB signaling, and induces oxidative stress and DNA damage, thereby preserving a chronic inflammatory state favorable to neoplastic development [60,61]. Additionally, *pks^+^ Escherichia coli* strains produce colibactin, a potent genotoxin capable of generating DNA cross-links and double-strand breaks, leading to genomic instability in colonocytes. This mechanism is supported by the presence of the characteristic mutational signature SBS88 identified in human colorectal cancer genomes [60,62]. The convergence of these stressors compromises epithelial barrier integrity, amplifies oxidative and genotoxic damage, and promotes the emergence of transformed epithelial cell populations. Therefore, via sustained metabolic, inflammatory, and signaling perturbations, the microbial community eases the formation, maintenance, and expansion of local cancer stem cell populations, thereby functioning as a persistent pro-carcinogenic force within the colonic niche. Collectively, these processes support a model in which gut microbial dysbiosis, together with the enrichment of oncogenic bacterial species, possibly acts as a critical ecological and biochemical driver of colorectal cancer (CRC) initiation and progression (Figure 1) [62].

### 5.2. Gastric Cancer

**Figure 2:**
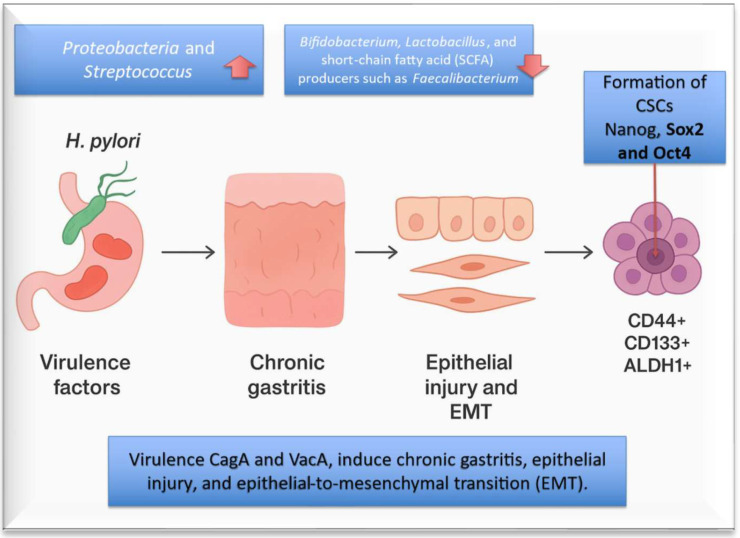
Factors that alter the composition of the human gastric microbiota. Many factors have been identified that play a predominant role in altering the composition of the human gastric microbiota. Among these, the most important remains *H. pylori*, responsible for gastric mucosal inflammation and oncogenic epithelial to mesenchymal transformation (EMT) through its main virulent factors such as CagA and VacA.

*Helicobacter pylori* is classified by the WHO as a Group I carcinogen. *H. pylori* infection is associated with a significant decrease in the overall diversity of gastric microbiota, often accompanied by an increase in inflammation-associated bacteria, such as *Proteobacteria* and *Streptococcus*, and a decrease in beneficial bacteria like *Bifidobacterium, Lactobacillus*, and short-chain fatty acid (SCFA) producers such as *Faecalibacterium* [63]. Its virulence factors, especially *Fusobacterium nucleatum* “interacting with N-nitroso compounds (NOCs), induce chronic gastritis, epithelial injury, and epithelial-to-mesenchymal transition (EMT). Following translocation into epithelial cells via the type IV secretion system, CagA undergoes tyrosine phosphorylation and activates a series of key oncogenic signaling pathways, including MAPK/ERK, PI3K/AKT, NF-κB, and Wnt/β-catenin. Activation of these cascades progressively disrupts epithelial cell polarity, promotes aberrant proliferation, and induces genomic instability [52,64]. In parallel, VacA, through its pore-forming properties and mitochondrial-targeting activity, contributes to apoptosis resistance, immune modulation, and sustained impairment of mucosal barrier permeability [65,66]. The EMT is achieved through upregulation of transcriptional repressors such as Snail, Slug, and Zeb1, endowing the formation of gastric epithelial cells with stem-like features, and therapy-resistant properties, contributing to the formation of decisive gastric cancer stem cells (CSCs) (CD44^+^, CD133^+^, ALDH1^+^) as well as stemness-associated key gene regulators, such as Nanog, Sox2 and Oct4 (Figure 2) [67,68]. These CSC-like populations enhance tumor-initiating capacity and could be potentially considered a key biological link between chronic *H. pylori* infection, early pre-neoplastic lesions, and ultimate malignant transformation supporting invasion, immune evasion, and tumor initiation [69,70].

### 5.3. Cervical and Uterine (Endometrial) Cancer

**Figure 3:**
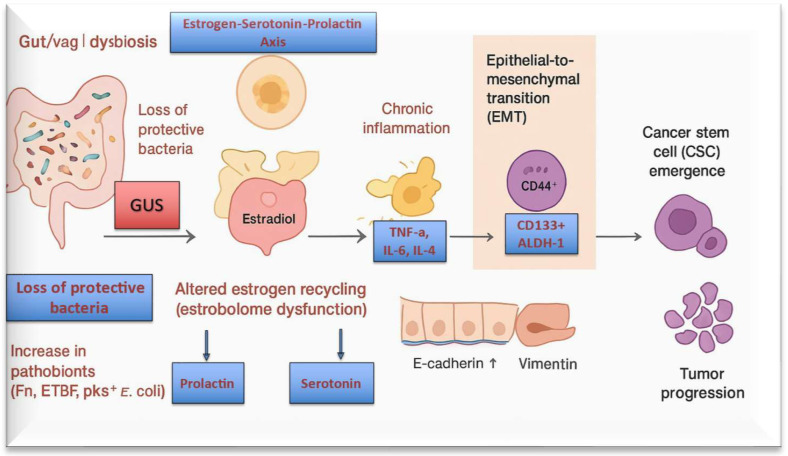
Schematic representation of the modulation of uterine function through the hormone-gut and urogenital microbiota axis. The secretion of GUS by intestinal bacteria converts conjugated estrogens into deconjugated estrogens in the gastrointestinal tract. The deconjugated estrogens are reabsorbed from the intestine and released into the bloodstream, facilitating the entry of estrogens into the uterus, where they exert their downstream effects.

Emerging evidence links endometrial cancer with vaginal dysbiosis, resulting in chronic inflammation and altered estrogen, prolactin and serotonin metabolism. Recent studies and mechanistic reviews highlight the regulatory role of gut microbial GUS in estrogen-dependent diseases [71,72,73]. Here it is necessary to introduce the importance of estrebolome which is the set of intestinal microbial genes that plays a crucial role in the homeostasis of the uterine/cervix microenvironment. The concept of the estrobolome refers to the collection of microbial genes involved in estrogen metabolism, primarily through β-glucuronidase (GUS) activity, which deconjugates excreted estrogens and enables their enterohepatic reabsorption [74,75]. Although the estrobolome has been traditionally associated with the gut microbiota, vaginal and uterine microbial communities can also influence local estrogen deconjugation and signaling pathways and, indirectly, the systemic estrogen pool via inflammatory signaling, hepatic metabolism, and broader microbial ecosystem interactions. Increased local and systemic estrogen exposure driven by elevated microbial GUS activity may support the development of estrogen-dependent endometrial tumors (Figure 3) [76,77].

Elevated GUS activity has been associated with increased systemic estrogenic tone and reduced progesterone excretion, potentially favoring endometrial hyperplasia and carcinoma in predisposing contexts such as obesity, chronic anovulation, metabolic syndrome, genetic susceptibility, and chronic depressive states [78,79]. Furthermore, this microbiota appears to influence the production of tryptophan-derived metabolites and signaling molecules that modulate serotonin synthesis both locally in the gut and systemically. Through vagal and cytokine-mediated pathways, these signals may directly affect the hypothalamic–pituitary axis, suggesting a potential indirect influence on prolactin secretion and proliferative signaling within the endometrium. Although direct human evidence remains limited, this mechanistic framework is biologically plausible and supported by emerging experimental data [79,80].

Eventually, these mechanisms may help explain how uterine and cervical dysbiosis weakens local immune surveillance. This condition is characterized by the loss of Lactobacillus dominance and the overgrowth of anaerobic species such as *Gardnerella vaginalis*, *Atopobium vaginae*, and *Chlamydia trachomatis*, thereby facilitating the persistence and entry of high-risk oncogenic viruses, including human papillomavirus (HPV), cytomegalovirus (CMV), and Epstein–Barr virus (EBV) [79,80,81]. Inflammatory cytokines and microbial-derived enzymes contribute to epithelial damage, promote viral integration into the host genome, and impair local immune defenses.

*Chlamydia trachomatis*, in particular, disrupts apoptotic pathways and p53 signaling, synergizing with HPV oncogenes (E6 and E7) to drive neoplastic transformation. This cooperative interaction favors the reprogramming of local epithelial stem and progenitor cells toward a cancer stem cell phenotype, characterized by the expression of markers such as CD24, CD166, EpCAM, CD44^+^, CD133^+^, and ALDH1^+^ [79,80,81].

### 5.4. Prostate Cancer

Although direct experimental validation of an estrobolome-like system within the prostate microenvironment is currently lacking, and the precise route of microbial modulation of estrogen availability remains to be defined, the prostate, similarly to the uterine and vaginal districts, constitutes a highly specialized and dynamic microenvironment in which microbial, hormonal, and immune signals are likely to intersect [81,82,83]. Emerging evidence indicate that the prostate harbors its own microbiota, and that alterations in this microbial ecosystem profoundly influence inflammation, epithelial integrity, hormone metabolism, and immune signaling [84,85]. Prostate dysbiosis has been associated with an enrichment of pro-inflammatory bacteria, including *Escherichia coli*, *Enterococcus faecalis*, *Propionibacterium acnes*, and *Fusobacterium nucleatum*, accompanied by a reduction in protective commensals [86,87]. This imbalance is linked to increased lipopolysaccharide (LPS) burden, disturbances in short-chain fatty acid profiles, and the accumulation of microbial metabolites capable of modulating host signaling pathways [86,87,88]. Given the prostate’s strong hormone dependence, dysbiosis has been proposed to affect the local hormonal environment through multiple mechanisms that remain incompletely characterized [89,90]. Certain bacterial taxa express enzymes capable of modifying steroid hormones; for example, microbial metabolism may contribute to the local conversion of testosterone to dihydrotestosterone (DHT) or estradiol (E2) [90,91,92]. Although direct experimental evidence within the prostate is limited, these observations raise the possibility that microbiota-driven steroid modulation may influence androgen receptor (AR) activity, even under conditions of systemic androgen deprivation, thereby potentially contributing to AR reactivation and the emergence of castration-resistant phenotypes [92,93].

In parallel, dysbiosis has been hypothesized to influence local estrogen metabolism through an estrobolome-like mechanism, analogous to that described in the intestinal tract. Such alterations may shift the balance between estrogen receptor alfa and beta (ERα and Erβ) signaling within the prostatic microenvironment, favoring ERα-associated proliferative and inflammatory responses while attenuating ERβ-mediated tumor-suppressive effects [92,93]. Although this model remains speculative, it is supported by converging evidence linking estrogen signaling, chronic inflammation, and prostate carcinogenesis.

**Figure 4:**
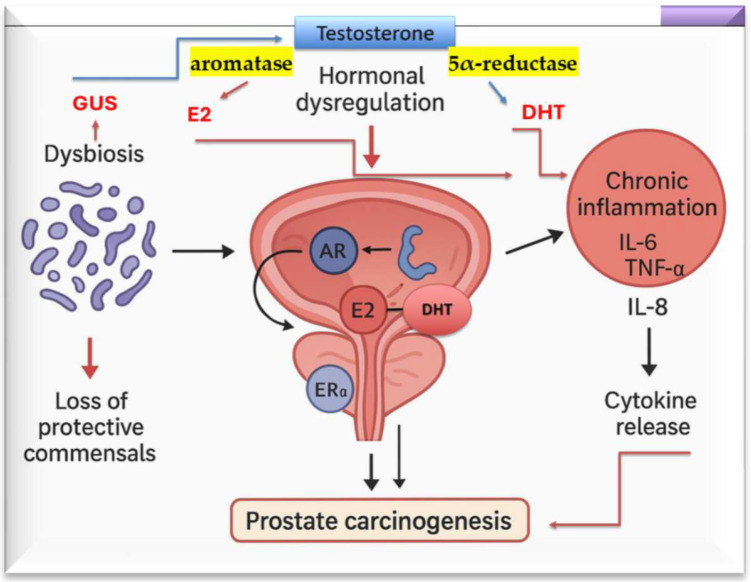
The secretion of microbial GUS by intestinal bacteria promotes the deconjugation of glucuronidated sex steroids, including estrogens and testosterone (testosterone glucuronide), restoring their biologically active forms within the gastrointestinal tract. This process increases the enterohepatic recirculation and systemic availability of free sex hormones, thereby influencing host endocrine balance. Elevated levels of deconjugated testosterone may subsequently be metabolized via aromatase into estrogens or via 5α-reductase into dihydrotestosterone (DHT) in peripheral tissues, contributing to hormone-dependent signaling pathways involved in inflammation, epithelial proliferation, and carcinogenesis.

Collectively, these observations suggest a dynamic hormone–bacteria crosstalk in which dysbiosis-associated chronic inflammation may further perturb hormonal signaling through cytokine-mediated AR phosphorylation, induction of AR splice variants such as AR-V7, and altered intraprostatic steroidogenesis within stromal cells [93,94]. Together, these processes generate a biochemical microenvironment permissive to tumor initiation and progression. This triangular interplay—microbial dysbiosis, hormonal dysregulation, and chronic inflammation—thus represents a plausible ecological and biochemical contributor to prostate carcinogenesis and disease progression (Figure 4) [95].

### 5.5. Bladder Cancer

These sex-specific microbial patterns suggest distinct immunological and metabolic microenvironments within the urinary tract, which may differentially influence susceptibility to inflammation, infection, and neoplastic transformation. The predominance of *Lactobacillaceae* in women likely contributes to mucosal immune homeostasis, whereas the greater heterogeneity observed in men may reflect increased variability in host–microbe interactions [96,97].

Age-related remodeling of the urinary microbiome may create a permissive microenvironment for carcinogenesis by favoring chronic low-grade inflammation, altered metabolic signaling, and diminished immune surveillance. In this context, loss of microbial homeostasis compromises urothelial barrier function and immune–epithelial crosstalk, thereby facilitating tumor initiation and progression through overlapping and self-reinforcing pathways [96,97].

Chronic inflammation is a central driver of urothelial carcinogenesis. Several urinary bacteria induce sustained inflammatory responses through pattern recognition receptor activation. Species capable of engaging Toll-like receptor 9 (TLR9) have been shown to shift the mucosal environment toward a pro-tumorigenic, rather than protective, immune phenotype [98,99]. The activation of these pathways can promote urothelial proliferation, increase oxidative stress, and stimulate the release of cytokines such as IL-6 and IL-8, all of which contribute to a microenvironment that favors strong inflammatory responses that may be persistent during the years [47,100].

Bladder tumor tissues have been shown to harbor increased abundance of bacterial genera such as *Fusobacterium*, *Actinomyces*, and *Corynebacterium* recognized for their pathogenic potential in mucosal tissues, and their presence correlates with structural disruption of the urothelium [101,102]. A key mechanism by which urinary microbes contribute to carcinogenesis is their metabolic activity. Several taxa possess nitrate-reducing capacity, leading to the formation of NOCs, a well-established class of urothelial carcinogens. For instance, *Fusobacterium nucleatum*, known for its capacity to adhere to epithelial cells and disrupt junctional integrity, has been implicated in both colorectal and bladder cancers. These effects are mediated primarily by its adhesins FadA and Fap2: FadA binds E-cadherin, triggering β-catenin signaling and the transcription of oncogenic and stemness-related genes, while Fap2 engages immune inhibitory receptors such as TIGIT and activates TLR4–MYD88–NF-κB signaling, thereby promoting local inflammation and immune evasion [47,100,101,102].

Comparable mechanisms have been implicated in colorectal cancer, where NOC-associated DNA damage may accelerate disease progression [101,102]. These effects involve the direct interaction with and modulation of junctional proteins, particularly E-cadherin, leading to structural disruption of epithelial adhesion complexes. Such perturbations increase mucosal permeability, expose basal epithelial layers to luminal carcinogens, and facilitate inflammatory cell infiltration, thereby promoting tumor initiation and progression [55,103]. These metabolites can directly damage DNA, induce mutations, and accelerate genomic instability [104,105]. Beyond nitrosamines, bacterial metabolism of dietary components, host-derived substrates, and xenobiotics may generate additional carcinogenic intermediates. Importantly, such metabolites may exert effects not only in the bladder but also at distant sites, suggesting that urinary dysbiosis could participate in systemic carcinogenic processes [104,105].

### 5.6. Breast Cancer

Through enhanced GUS-mediated deconjugation of estrogens, dysbiotic gut microbiota increase systemic estrogen bioavailability, thereby sustaining estrogen receptor-dependent proliferative signaling in breast tissue. In parallel, dysbiosis-associated immune dysregulation and chronic inflammation may further support tumor initiation and progression. This concept is supported by experimental evidence indicating that probiotic-conditioned media selectively impairs the viability of estrogen receptor-positive (ER^+^) breast cancer cells and differentially modulates mitochondrial metabolic activity in non-malignant epithelial cells, potentially through β-glucuronidase (GUS)-mediated mechanisms [106,107]. These enzymes deconjugate estrogens excreted in bile, enabling their reabsorption into the bloodstream via enterohepatic circulation [106,107].

This mechanism becomes especially relevant in postmenopausal women, in whom endogenous estrogen production is physiologically reduced. In this context, the delicate interplay among estrogen, prolactin, and serotonin is often destabilized, influencing peripheral hormone metabolism and microbial reactivation of conjugated estrogens [108,109,110].

Because estrogen receptor-positive (ER^+^) breast cancers account for approximately 70% of all breast malignancies, the estrobolome is of considerable interest for its capacity to modulate systemic estrogen levels and thus affect cancer risk and progression. Certain bacteria—including *Enterococcus faecalis* and *Bacteroides fragilis*—produce high levels of GUS, increasing circulating bioactive estrogens that bind to estrogen receptors in breast tissue and may enhance proliferation of cancer stem-like cells [111,112,113].

Beyond hormone metabolism, dysbiosis influences systemic inflammation, oxidative stress, and immune signaling, all of which can alter the breast microenvironment and create conditions favorable to tumor initiation or progression. Notably, microbial DNA and viable bacteria such as *Enterococcus faecalis* and *Bacteroides fragilis* have been detected within breast tumor tissues, suggesting the possibility of local colonization and a more direct role in tumor biology [114,115].

## 6. Dysbiosis-Mediated Generation of Abnormal Stem Cells as the First Hit for Cancer via Mitochondrial Subversion

Persistent dysbiosis may act as an initiating “first hit” in carcinogenesis, establishing a field of epithelial vulnerability rather than directly inducing malignant transformation. By imposing chronic microenvironmental stress, microbial imbalance disrupts epithelial integrity, stem-cell homeostasis, and regenerative signaling, thereby predisposing large mucosal areas to subsequent oncogenic events [116]. This altered ecosystem is characterized by expansion of harmful microbial taxa, depletion of protective commensals, and breakdown of microbe–host communication pathways that normally maintain epithelial renewal [117,118]. Within this pre-neoplastic field, enhanced microbial enzymatic activity—such as the increased hydrolysis of O-, N-, and N^+^-glucuronide metabolites in human feces as described by Zhang et al., can lead to localized reactivation of potentially genotoxic compounds. Such persistent biochemical re-exposure may amplify inflammatory signaling, destabilize stem-cell niches, and promote field cancerization, thereby lowering the threshold for secondary genetic or epigenetic “hits” that ultimately drive malignant progression [119].

A central mechanistic axis underlying these processes involves the destabilization of both mitochondrial function and mito–nuclear communication within resident stem and progenitor cell populations. Microbial-derived metabolites, including genotoxins, ROS-inducing factors, and pro-inflammatory mediators, may eventually compromise DNA repair capacity, remodel stem cell signaling networks, and drive metabolic reprogramming toward oxidative stress or Warburg-like glycolytic phenotypes [118,119]. These convergent alterations increase mutational vulnerability and bias stem cells toward aberrant proliferative and survival pathways, thereby facilitating malignant transformation.

Dysbiosis also perturbs the local immunoendocrine environment. Hormones such as E2, testosterone, prolactin, pregnelolone and serotonin regulate stem-cell cycling, mitochondrial dynamics, redox balance, and epithelial repair. Alterations in microbial composition influence hormone metabolism, degradation, and receptor signaling—for example, through microbial GUS, mediated deconjugation of estrogen metabolites or via modulation of tryptophan, serotonin pathways [120,121,122]. Perturbed hormone signaling increases stem-cell susceptibility to mitochondrial stress and reduces resilience to environmental insults.

Emerging evidence shows that mitochondrial dysfunction is associated with increased microbial GUS activity, particularly within immune cells such as macrophages and T lymphocytes. Mitochondrial stress promotes enhanced lactate production and extracellular acidification, conditions that favor microbial GUS enzymatic activity and the deconjugation of glucuronidated hormones, xenobiotics, and potential carcinogens in the gut and peripheral tissues as shown in Figure 3 and Figure 4 [123,124]. The resulting increase in biologically active compounds further perturbs endocrine signaling and exacerbates mitochondrial instability, thereby establishing a self-reinforcing loop of metabolic and endocrine stress [123,124].

Persistent dysbiosis contributes to mitochondrial impairment through multiple converging mechanisms, including: (i) disruption of redox homeostasis with excessive ROS generation; (ii) defective mito–nuclear communication; (iii) metabolic reprogramming toward aerobic glycolysis or inefficient oxidative phosphorylation; and (iv) reduced mitochondrial biogenesis together with impaired mitochondrial DNA (mtDNA) repair capacity [125,126]. Collectively, these alterations compromise stem and progenitor cell resilience and favor the emergence of metabolically reprogrammed progenitors with early cancer stem cell-like features, characterized by enhanced survival under inflammatory, hypoxic, and nutrient-restricted microenvironmental conditions [126,127].

As previously discussed, aging represents a major potential confounder in these processes and was therefore included as an independent variable in multivariable analyses. However, the observation of reduced total androgen levels in specific populations, such as shift workers and elderly individuals, does not exclude a potential association between circadian disruption, hormonal imbalance, and prostate cancer risk [127]. Epidemiological studies evaluating the relationship between circulating hormones, including testosterone, dihydrotestosterone (DHT), pregnenolone, and estradiol (E2), and prostate cancer have yielded conflicting results, with some reporting increased risk at higher testosterone levels and others showing no significant association [128,129].

Notably, circulating levels of testosterone, estradiol (E2), and dihydrotestosterone (DHT) exhibit an inverse correlation with prostate volume in aging males [128]. At the same time, the incidence of prostate cancer increases with age, a process typically accompanied by a progressive decline in circulating testosterone levels together with increased intraprostatic activity of aromatase and 5α-reductase [128,129,130]. Collectively, these observations highlight the complex and non-linear interactions among aging, systemic and local androgen–estrogen signaling, mitochondrial function, and prostate carcinogenesis [131,132].

## 7. Conclusions

Beyond its conceptual implications, this framework, centered on the interplay between microbial dysbiosis, hormonal regulation, and stem cell dynamics, provides strategic guidance for future clinical and translational research. Positioning aging and the microbiome as upstream and modifiable determinants of cancer susceptibility highlights novel intervention opportunities that extend beyond conventional cytotoxic strategies or mutation-focused approaches, as well as stem cell-based and hormone interventions that are not integrated with microenvironmental modulation and have shown limited efficacy.

This emerging multi-disciplinary approach may eventually include the targeted modulation of microbial enzymatic functions, such as inhibition of GUS activity to prevent the local reactivation of pro-carcinogenic metabolites, as well as the development of mitochondria-targeted prebiotics or rationally designed microbial consortia aimed at restoring epithelial bioenergetic competence, redox homeostasis, and regenerative capacity. In parallel, cell-based and cell-free platforms, including stem cells and their derived extracellular vesicles or exosomes, may be viewed as biologically informed antitumor vectors capable of modulating the tumor microenvironment, delivering metabolic, immunoregulatory, and paracrine signals, and counteracting dysbiosis-induced niche conditioning. Within this reconstructive vision, hormone replacement therapy may also be leveraged as a modulatory axis, contributing to the restoration of epithelial homeostasis, stem cell regulation, and microenvironmental balance.

Overall, these considerations illustrate how a microbiome- and microenvironment-focused model of carcinogenesis can guide the development of preventive, diagnostic, and therapeutic strategies targeted at the earliest and potentially reversible stages of cancer growth. In conclusion, microbial dysbiosis acts as an upstream ecological disruptor, reshaping aging-related processes, including mitochondrial bioenergetics, hormonal signaling, and stem cell homeostasis, long before a malignant phenotype becomes clinically detectable. Elucidating the complex, bidirectional interactions among the microbiota, hormones, and mitochondria may reveal early determinants of tissue vulnerability, refine cancer risk stratification, and identify actionable targets for timely, microenvironment-focused preventive intervention.

## Figures and Tables

**Table 1:** Mechanistic Interactions Linking Dysbiosis, Hormones, and Mitochondrial Dysfunction in Early Cancer Initiation.

Mechanistic Domain	Dysbiosis-Induced Effect	Consequence for Stem Cells
Microbial imbalance	Loss of commensals; overgrowth of pathobionts	Reduced epithelial integrity; increased inflammatory signaling
Microbial metabolites and genotoxins	ROS inducers, DNA-damaging toxins	Impaired DNA repair; increased mutational load
Mitochondrial function	Mitochondrial stress, impaired OXPHOS, and apoptotic mechanisms	Rise in ROS; defective mito–nuclear signaling
Hormone metabolism	Altered estrogen/testosterone/prolactin/cortisol handling; changes in serotonin pathways	Dysregulated proliferation; mitochondrial modulation
Lactate–GUS axis	Lactate-driven acidification increases macrophage GUS activity	Reactivation of estrogen metabolites and toxins → stem-cell destabilization
Metabolic shifts	Increased glycolysis; decreased mitochondrial efficiency	Vulnerability to transformation; pre-malignant metabolic reprogramming
Systemic feedback loop	Dysbiosis ↔ mitochondrial dysfunction ↔ endocrine disruption	Creation of a pro-oncogenic niche

## Data Availability

No new data were created or analyzed in this study. Data sharing is not applicable to this article.

## Abbreviations

GUS	β-glucuronidase
STAT1–3	Signal Transducer and Activator of Transcription 1–3
SMAC/DIABLO	Second Mitochondria-Derived Activator of Caspases/Direct IAP-Binding Protein with Low pI
HIF-1	Hypoxia-Inducible Factor-1
SCFAs	short-chain fatty acids
CAZymes	microbiota-encoded carbohydrate-active enzymes
(FXR)	nuclear farnesoid X receptor
UTI	urinary tract infection
ROS	reactive oxygen species
ETBF	enterotoxigenic *Bacteroides fragilis*
BFTs	*Bacteroides fragilis* toxins
EMT	epithelial to mesenchymal transformation
HPV	Human Papillomavirus
CMV	Cytomegalovirus
EBV	Epstein–Barr Virus
DHT	dihydrotestosterone
E2	Estradiol
AR	androgen receptor
Erα	Estrogen receptor alpha
Erβ	estrogen receptor beta
TLR9	Toll-like receptor 9
